# Doxorubicin-induced renal inflammation in rats: Protective role of *Plantago major*

**Published:** 2018

**Authors:** Nazanin Entezari Heravi, Sara Hosseinian, Zohreh Naji Ebrahimi Yazd, Mohammad Naser Shafei, Alireza Ebrahimzadeh Bideskan, Samira Shahraki, Zahra Samadi Noshahr, Fatemeh Motejadded, Farimah Beheshti, Reza Mohebbati, Soghra Parhizgar, Abolfazl Khajavi Rad

**Affiliations:** 1 * Department of Physiology, School of Medicine, Mashhad University of Medical Sciences, Mashhad, Iran *; 2 * Neurogenic Inflammation Research Center, Mashhad University of Medical Sciences, Mashhad, Iran*; 3 *Division of Neurocognitive Sciences, Psychiatry and Behavioral Sciences Research Center, Mashhad University of Medical Sciences, Mashhad, Iran*; 4 *Department of Anatomy and Cell Biology, School of Medicine, Mashhad University of Medical Sciences, Mashhad, Iran*; # * Co-first author*

**Keywords:** Plantago major, Doxorubicin, Vitamin E, Dexamethasone, Inflammation

## Abstract

**Objective:**

The aim of the present study was to evaluate the possible protective effect of *Plantago major* (*P. major*) extract against doxorubicin (DXR)-induced renal inflammation in rats.

**Materials and Methods:**

80 male albino rats were randomly divided into 8 groups as follows: control, DXR, Ext (extract) 600, Ext1200, dexamethasone+DXR, vitamin E+DXR, Ext600+DXR*, *and Ext1200+DXR. Duration of the study was 35 days and DXR was intravenously injected on the 7^th^ day of the experiment. Tumor necrosis factor-alpha (TNF-α) production and monocyte chemoattractant protein-1 (MCP-1) expression levels were assessed in the left kidney. Serum creatinine concentration and osmolarity were determined on the 1^st^, 14^th^, 21^st^, 28^th^ and 35^th^ days of the experiment.

**Results:**

DXR caused a significant increase in renal expression of MCP-1 and TNF-α production compared to control animals. Administration of dexamethasone, vitamin E and *P. major* extract significantly improved the expression of these inflammatory mediators compared to DXR group. Compared to day 1 in DXR group, serum osmolarity showed a significant increase on days 21, 28 and 35. Also, on these days, serum osmolarity in DXR group was significantly higher than that on the same days in control group. In Vit E+DXR and Ext 1200+DXR groups, there was no significant changes in serum osmolarity among different days of the study. However, in these groups, serum osmolarity on days 21, 28 and 35 showed a significant decrease compared to the same days in DXR group.

**Conclusion:**

Present results suggest that hydroethanolic extract of *P. major* protected renal tissue against DXR–induced renal inflammation.

## Introduction

Doxorubicin (DXR), also known as adriamycin, is a widely used antineoplastic chemotherapeutic that is used for the treatment of different neoplastic conditions including uterine sarcoma, acute lymphoblastic leukemia (ALL), and multiple myeloma, as well as breast, liver and lung cancers (Katzung et al., 2012 ▶). Some of molecular underllying mechanisms proposed for DXR are intercalation with DNA and inhibition of nucleic acid synthesis, as well as reactive oxygen species (ROS) generation (Cummings et al., 1991 ▶). In spite of DXR's high efficacy in the treatment of tumors, its clinical use is highly restricted due to severe side effects; one of the most important adverse effect of DXR is nephropathy. Nephropathy is an important cause of nephrotic syndrome, a combination of proteinuria, low blood albumin level, hyperlipidemia and edema (Orth and Ritz, 1998 ▶). DXR-induced nephropathy is a widely used experimental model in rodents to produce experimental proteinuric nephropathy (Okuda et al., 1986 ▶). Although the exact mechanisms underlying DXR-induced nephropathy are not fully understood, the role of oxidative stress and inflammation has been demonstrated in this model (Szalay et al., 2015 ▶). ROS generation is assumed to have a central role. It has been shown that DXR injection leads to a marked increase in renal lipid peroxidation and decreases in renal total antioxidant capacity and antioxidant enzyme activity in rats (Mohebbati et al., 2016 ▶). Also, DXR administration leads to prominent tubulointerstitial inflammation with marked lymphocytes and macrophages infiltration. This local inflammation may be due to production of cytokines and growth factors like tumor necrosis factor-α (TNF-α), transforming growth factor-β (TGF-β) and chemotactic factors (monocyte chemoattractant protein-1 (MCP-1)) in response to proinflammatory mediators and infiltrated cells (Szalay et al., 2015 ▶). Nuclear factor κB (NF-κB) is a transcription factor that controls cytokine production and many other inflammatory genes expression. It has been demonstrated that inhibition of NF-κB reduced tubulointerstitial injury in DXR-induced nephropathy (Yamashita et al., 2017 ▶). Increasing data suggest that modifying inflammatory and oxidative pathways may alleviate DXR-induced renal damage. *Plantago major*, a flowering plant belonging to the family Plantaginaceae, is one of the most commonly grown medicinal plants throughout the world (Samuelsen, 2000 ▶). *P. major* contains different biologically active compounds including flavonoids, terpenoids, alkaloids, lipids, and polysaccharides (Jamilah et al., 2012 ▶) that possess anticancer, anti-ulcerogenic, immunomodulatory, antimicrobial, anti-inflammatory and antioxidant properties (Miraj, 2016 ▶). The aim of the present study was to evaluate the possible protective effects of *P. major* against DXR-induced renal inflammation in rats.

## Materials and Methods

**Extract preparation**

*P. major* whole plant was well-dried and then grounded to powder. The powder was extracted using a Soxhlet extractor using ethanol (70%). The extract was concentrated in a rotary evaporator. It was then kept at 4^oC^ prior to use. 

**Chemicals**

DXR was purchased from Ebewe Pharma Company (Austria). Vitamin E (Vit E) and dexamethasone (DEX) were obtained from Osve Pharmaceutical Company (Iran). 

**Animals**

Eighty male Albino Wistar rats weighing 200-250 g were obtained from the Animal House of the School of Medicine, Mashhad University of Medical Sciences, Mashhad, Iran. The animals were housed at room temperature (25±1 ^o^C) on a regular 12 hr/12 hr light/dark cycle with free access to food and water *ad libitum*. All experiments were approved by ethics committee of Mashhad University of Medical Sciences, Mashhad, Iran.

**Experimental design**

In the present study, animals were randomly divided into 8 groups of 10 rats as follows: 1) Control: this group received an injection of normal saline via intravenous tail injection (i.v.) on the 7^th^ day of the experiment, 2) DXR: this group received an injection of DXR (5 mg/kg, i.v.) on the 7^th^ day of the experiment, 3) Ext- 600: this group received *P. major *extract (600 mg/kg, in drinking water) for 5 consecutive weeks and injected with normal saline on the 7^th^ day of the experiment, 4) Ext-1200: this group received *P. major *extract (1200 mg/kg, in drinking water) for 5 consecutive weeks and injected with normal saline on the 7^th^ day of the experiment, 5) DEX+DXR: this group received an injection of DEX (0.9 mg/kg i.p.) for 6 consecutive days before injection of DXR, and for 2 weeks after that, every other day, 6) Vit E+DXR: this group received vitamin E (100 mg/kg, in drinking water) for 5 consecutive weeks and injected with DXR on the 7^th^ day of the experiment, 7) Ext 600+DXR: this group received *P. major *extract (600 mg/kg, in drinking water) for 5 consecutive weeks and injected with DXR on the 7^th^ day of the experiment, and 8) Ext 1200+DXR: this group received *P. major *extract (1200 mg/kg, in drinking water) for 5 consecutive weeks and injected with DXR on the 7^th^ day of the experiment.

Blood samples were collected from the orbital sinus on the 1^st^, 14^th^, 21^st^, 28^th^ and 35^th^ days of the experiment. Blood samples were centrifuged at 3000 rpm for 15 min, and serum was stored at -20 °C until used. Serum concentration of creatinine was measured by Convergys®100 Biochemistry Analyser using the commercial kit (Pars Azmoon Company, Iran). Serum osmolarity was determined by a cryoscopic osmometer (Osmomat 030, Germany). Four weeks after DXR injection, animals were killed and the left kidneys were removed and divided into two halves. One half was fixed in 10% formalin for the assessment of immunolocalization of MCP-1. The other half was kept at -80°C for determination of TNF-α concentration.

**Immunohistochemical examinations**

The fixed kidney tissues were embedded in paraffin and cut into 5-μm thick sections. Then, tissue sections were deparaffinized and incubated with MCP-1 antibody (rabbit polyclonal anti-MCP-1, Biorbyt, UK; dilution 1:100) at 4°C overnight; then; samples were incubated with HRP- conjugated secondary antibody (goat polyclonal secondary antibody to rabbit IgG, Biorbyt, UK; dilution 1:100). The sections were finally incubated with diaminobenzidine (DAB) as the chromogen. Then, the slides were counterstained with hematoxylin and finally examined by light microscopy. Three blind examiners evaluated the intensity of staining using the following scoring system: 0=no staining, 1=weak, 2=moderate, and 3=severe (Zhang et al, 2010 ▶).

**Determination of TNF-α concentration**

Briefly, kidney tissue was homogenized and centrifuged at 6000 rpm for 10 min. The supernatant was collected and TNF-α level was measured using anti-rat TNF-α ELISA kit (IBL international, US) according to the manufacturer's instructions. 

**Statistical analysis**

All data were expressed as means±SEM. Comparison among groups was made using one-way ANOVA followed by LSD *post hoc* test. Intragroup comparisons were made using repeated measures. Differences were considered statistically significant when p<0.05.

## Results

**Figure 1:**
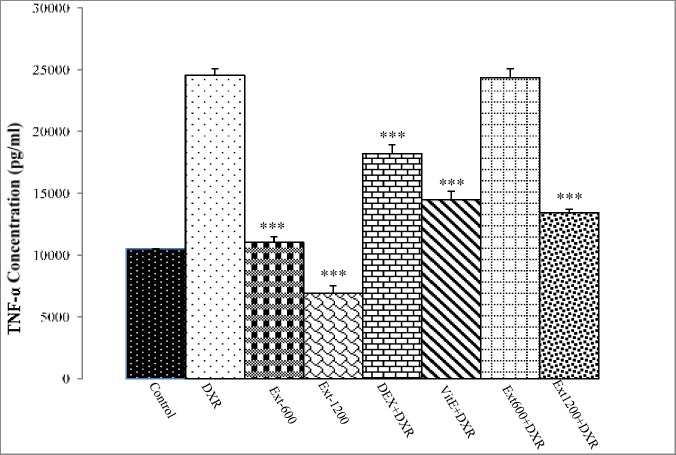
Renal TNF-α concentration in different experimental groups. Values are expressed as mean±SEM. *** p<0.001 indicates significant differences compared to the DXR group. DXR: doxorubicin; DEX: dexamethasone; Vit E: vitamin E; and Ext: *Plantago major *extract).

Renal TNF-α concentration significantly increased in DXR group compared to the control group (p<0.001). In Ext-600 and Ext-1200 groups, renal TNF-α production was significantly lower than that of the DXR group (p<0.001). Administration of DEX and Vit E significantly decreased renal TNF-α protein level compared to DXR-treated rats (p<0.001). Treatment of DXR-injected rats with *P. major* extract (1200 mg/kg) led to a significant reduction in renal TNF-α production compared to DXR group (Figure 1).

**Figure 2:**
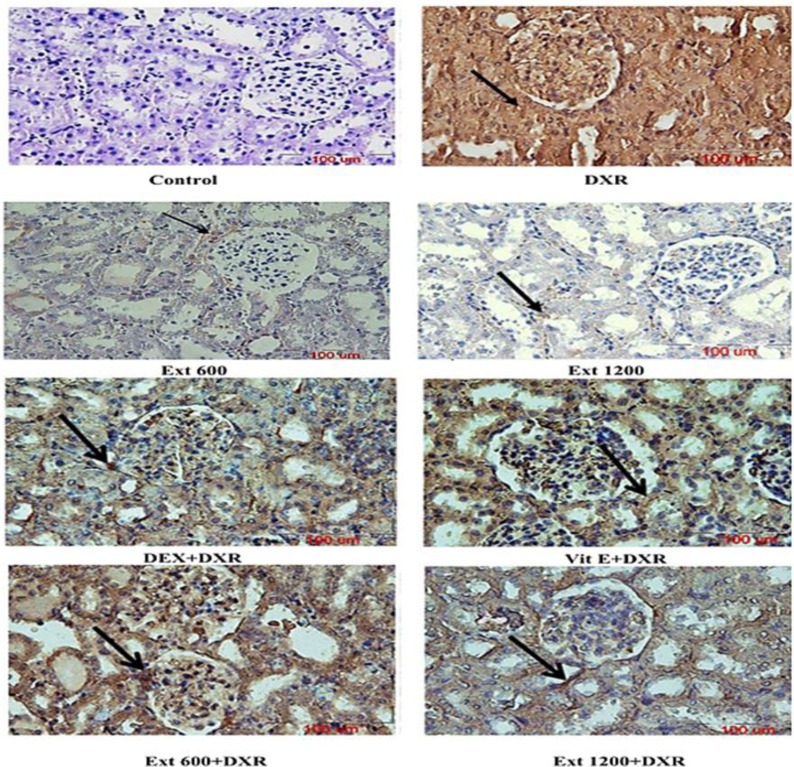
Photomicrographs showing immunolocalization of MCP-1 in the cortex (A) and medulla (B) of different experimental groups. Immunoreactivity is shown with arrows (magnification 400X; scale bar =100 µm). DXR: doxorubicin; DEX: dexamethasone; Vit E: vitamin E; and Ext: *Plantago major *extract.

Figure 2 shows renal immunohistochemical localization of MCP-1 protein and Table 1 demonstrates the estimation of MCP-1 immunoreactivity in renal tissue in different experimental groups. The results of immunohistochemical staining showed very weak expression of MCP-1 in renal tissues of the control animals, as well as, the Ext-600 and Ext-1200 groups. However, immunoreactivity for MCP-1 was strong in DXR-treated rats. 

Moderate MCP-1 immunoreactivity was observed in DEX+DXR and Vit E+DXR groups compared with DXR- treated rats. Also, the staining intensity of MCP-1 in kidney tissue decreased after administration of *P. major* extract at doses of 600 and 1200 mg/kg compared with DXR group (Figure 2 and Table 1). 

**Table 1:** The intensity scores of renal MCP-1 expression in different groups of animals.

	**Control **	**DXR **	**Ext-600 **	**Ext-1200 **	**DEX+DXR **	**Vit E+DXR **	**Ext 600+DXR **	**Ext 1200+DXR**
**MCP-1**	1	4	1	1	2	2	2	1-2

**Figure 3:**
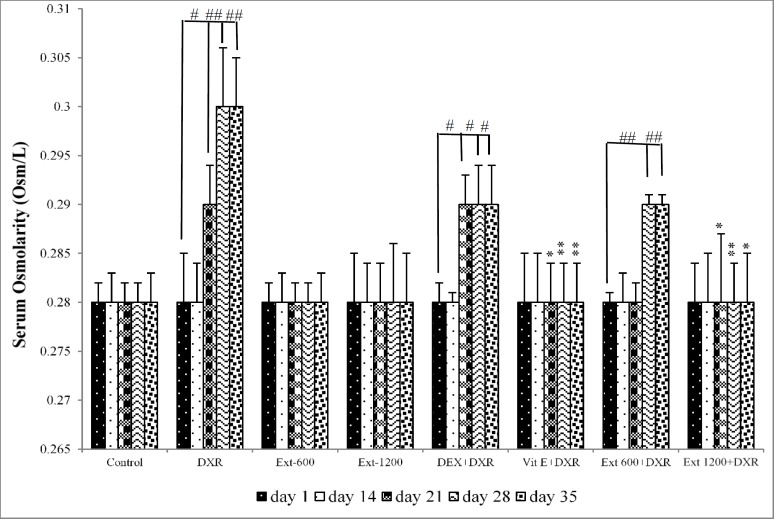
Serum osmolarity in all experimental groups. Values are expressed as mean±SEM.

*p<0.05 and **p<0.01 indicate significant differences compared to the results of the same days in DXR group.

^#^p<0.05 and ^##^p<0.01 indicate significant differences compared to day 1 in the same group.

(DXR: doxorubicin; DEX: dexamethasone; Vit E: vitamin E; and Ext: *Plantago major *extract).

**Figure 4:**
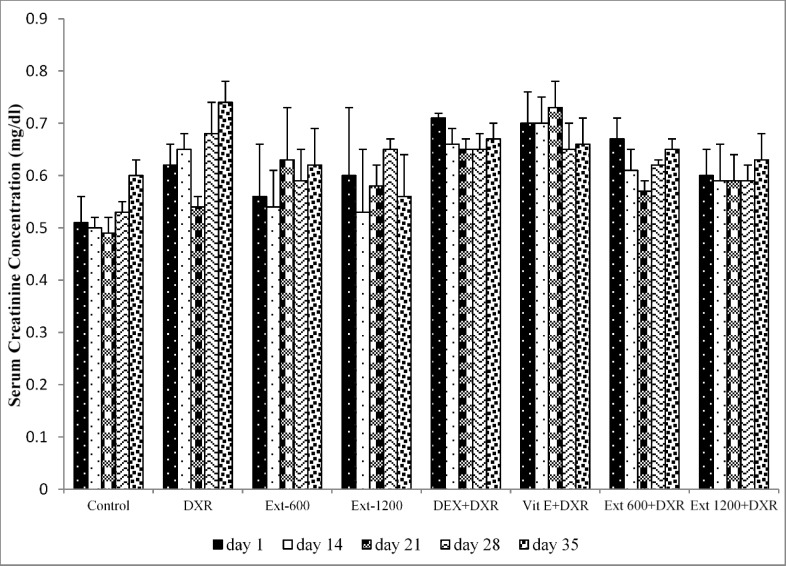
Serum creatinine concentration in all experimental groups. Values are presented as mean±SEM.

(DXR: doxorubicin; DEX: dexamethasone; Vit E: vitamin E; and Ext: *Plantago major *extract).

In the control, Ext-600 and Ext-1200 groups, there was no significant differences in serum osmolarity among different days of the experiment. However, compared to day 1 in DXR group, serum osmolarity showed a significant increase on days 21, 28 and 35 of the study (p<0.05, p<0.01 and p<0.01 respectively) (Figure 3). Additionally, on these days, serum osmolarity in DXR group was significantly higher than that of the control group on the same days (p<0.05, p<0.001 and p<0.01, respectively) (Figure 3). In the Vit E+DXR and Ext 1200+DXR groups, there was no significant differences in serum osmolarity among different days of the study. However, in the Vit E+DXR group, serum osmolarity on days 21, 28 and 35 showed a significant decrease compared to that of the DXR group on the same days (p<0.05, p<0.01 and p<0.05, respectively) (Figure 3). Furthermore, on these days, serum osmolarity in the Ext 1200+DXR group was also significantly lower than that of DXR-injected animals (p<0.05, p<0.01 and p<0.05, respectively) (Figure 3). However, serum creatinine concentration showed no significant difference among different days of the study (Figure 4). 

## Discussion

The results of the current study indicated that *P. major* extract significantly improves the renal expression of MCP-1 and TNF-α in DXR-injected rats. Various mechanisms have been suggested to explain DXR-induced nephropathy (Lee and Harris, 2011 ▶). Although it is commonly accepted that proteinuria is caused by injury to glomerular endothelial cells, glomerular basement membrane and podocytes, the exact mechanism of the DXR-induced nephropathy is not fully understood. In many experimental models of renal injury including cisplatin-induced nephrotoxicity (Hosseinian et al., 2016 ▶; Parhizgar et al., 2016 ▶), renal ischemia-reperfusion (Havakhah et al., 2014), unilateral ureteral obstruction (Hosseinian et al., 2017 ▶), and DXR-induced nephropathy (Mohebbati et al., 2016 ▶; Mohebbati et al., 2017 ▶), the role of oxidative stress and inflammation has been demonstrated. It has been postulated that free radical generation, lipid peroxidation and antioxidant enzymes inhibition are the main mechanisms underlying DXR nephrotoxicity (Szalay et al., 2015 ▶). In addition, DXR leads to a severe tubulointerstitial inflammation by local generation of cytokines and chemotactic factors in response to cellular injury, plasma protein filtration and glomerular inflammatory mediators (Rangan et al., 2000 ▶). The exact mechanisms underlying inflammatory action of DXR are unclear, but in recent investigations, the role of nuclear factor-kappa B (NF-κB), mitogen-activated protein kinases (MAPKs) signaling pathways and their interaction have been postulated (Kim et al., 2017 ▶). TNF-α, a pro-inflammatory cytokine, is produced after DXR administration by glomerular and tubular cells and extrinsic infiltrated inflammatory cells and acts through MAPKs and NF-κB signaling pathways (Neale et al., 1995 ▶). Activation of these pathways then, upregulates the expression of some inflammatory cytokines including TNF-α and MCP-1 (Hosseinian et al., 2017 ▶). In the present study, the nephropathy was created by a single dose intravenous injection of DXR (5 mg/kg). The results indicated a 2.33- fold increase in renal production of TNF-α, and a marked increase in renal expression of MCP-1 after DXR administration. These findings have also been reported by Rangan et al., (2000) ▶ and Benchetrit et al. (2001) ▶ who reported the upregulation of the above-mentioned inflammatory mediators following DXR administration (Rangan et al., 2000 ▶; Benchetrit et al., 2001 ▶). The present study revealed a significant decrease in TNF-α concentration in *P. major* extract-treated rats. Interestingly, the higher dose of the extract (1200 mg/kg) exerted more marked protection against TNF-α production compared to the lower dose (600 mg/kg). Also, a remarkable reduction was observed in MCP-1 expression in animals treated with *P. major* extract, showing that treatment with *P. major* extract attributes to improvement of inflammation. Our results also showed that these beneficial effects of *P. major* extract (1200 mg/kg) were comparable and even more pronounced than those of DEX and Vit E, which can confirm the anti-inflammatory and antioxidant effects of *P. major* extract. In the present work, serum osmolarity in DXR group showed a significant increase on the 21^st^, 28^th^ and 35^th^ days of the experiment. The exact mechanism of DXR effect on serum osmolarity has not been fully elucidated, but it seems that enhanced epithelial Na^+^/K^+^-ATPase activity, hypovolemia and sodium retention might be involved in this process (Deschenes and Doucet, 2000 ▶). Present results also showed that reducing effect of *P. major* extract on serum osmolarity in the Ext 1200+DXR group was more marked than those of the Ext 600+DXR group, which might indicate a dose-dependent improving effect for *P. major* extract on tubular transport processes. Meanwhile, based on our findings, serum creatinine concentration showed no significant differences among different experimental groups. This result possibly indicates that DXR administration with the dose used in this study, has no significant effect on renal processing of creatinine. 

In conclusion, the present study showed that *P. major* extract in a dose-dependent manner could improve renal inflammation associated with DXR that might partly be due to its antioxidant and anti-inflammatory actions. However, the exact mechanisms underlying the effects of *P. major* needs to be further evaluated.
